# Benchmarking Detachable-string Magnetic Capsule Endoscopy against the Right Standard for Early Esophageal Squamous Cell Cancer

**DOI:** 10.1055/a-2889-0416

**Published:** 2026-06-25

**Authors:** Ervin Toth, Anastasios Koulaouzidis

**Affiliations:** 1 Department of GastroenterologySkåne University Hospital, Lund UniversityMalmöSweden; 2 Department of Gastroenterology 37805 Pomeranian Medical University in SzczecinSzczecinPoland; 3 Department of Clinical Research 6174 University of Southern Denmark (SDU)SvendborgRegion SyddanmarkDenmark; 4 Department of Surgery 11286 SATC-C, Odense University Hospital & Svendborg HospitalSvendborgRegion SyddanmarkDenmark

## 

To the Editor,

We read with interest the exploratory study by Dai et al. evaluating detachable-string magnetically controlled capsule endoscopy (DS-MCCE) for esophageal lesions including early esophageal squamous cell carcinoma (ESCC). 1 The authors should be congratulated for testing a minimally invasive, patient-friendly platform in a clinically important setting. Their safety, comfort, and preference data are encouraging. We are concerned, however, that the diagnostic performance of DS-MCCE is positioned against an inappropriately weak benchmark.

In the Discussion, the authors state that the 66.67% sensitivity of DS-MCCE for early ESCC “approached” that of conventional white-light endoscopy (55%). 1 However, the relevant comparator in contemporary practice is image-enhanced endoscopy and not historical white-light imaging alone. In the authors’ own study, the reference examination included narrow-band imaging (NBI) in every patient, with Lugol chromoendoscopy applied to suspicious lesions. In the randomized trial cited by the authors, NBI detected superficial ESCC substantially more often than white-light imaging (97% vs. 55%). 2 Meta-analytic data likewise report per-patient sensitivities of approximately 88% for NBI and 92% for Lugol chromoendoscopy. 3 Against that standard, a sensitivity of 66.67% (95% CI 38.38–88.18%) cannot yet be regarded as competitive for screening.

The authors further suggest that missed lesions were mainly smaller or morphologically subtle. 1 However, their own Table 3 shows that the limitation is not confined to subcentimeter subtle abnormalities. One 2.0 cm high-grade dysplasia (HGD) lesion was interpreted as “Negative,” and another 1.0 cm HGD lesion was classified as reflux esophagitis. The latter is particularly concerning, because a false-benign label may reduce the perceived need for confirmatory endoscopy. An additional interpretive caution is that 27 examinations without suspicious findings on DS-MCCE were excluded from reference verification. 1 The authors appropriately acknowledge this limitation, but it means additional false-negatives could have remained undetected.

DS-MCCE should therefore be viewed as an interesting exploratory platform rather than a clinically benchmarked screening tool. The next step should be a prospective head-to-head study against contemporary image-enhanced endoscopy, with explicit accounting for false-negative and false-benign classifications. This is especially important because nonendoscopic alternatives are advancing rapidly; for example, AI-assisted sponge cytology has already shown high sensitivity and specificity at screening scale. 4


